# Cyclodextrin-Modified Nanomaterials for Drug Delivery: Classification and Advances in Controlled Release and Bioavailability

**DOI:** 10.3390/pharmaceutics13122131

**Published:** 2021-12-10

**Authors:** Daniel Andrés Real, Karen Bolaños, Josefina Priotti, Nicolás Yutronic, Marcelo J. Kogan, Rodrigo Sierpe, Orlando Donoso-González

**Affiliations:** 1 Laboratorio de Nanobiotecnología y Nanotoxicología, Departamento de Química Farmacológica y Toxicológica, Facultad de Ciencias Químicas y Farmacéuticas, Universidad de Chile, Santiago 8380544, Chile; daniel.real@ciq.uchile.cl (D.A.R.); k.bolaosjimenez@uandresbello.edu (K.B.); mkogan@ciq.uchile.cl (M.J.K.); 2 Advanced Center for Chronic Diseases (ACCDiS), Universidad de Chile and Pontificia Universidad Católica de Chile, Santiago 8380544, Chile; 3 Cellular Communication Laboratory, Program of Cellular and Molecular Biology, Center for Studies on Exercise, Metabolism and Cancer (CEMC), Facultad de Medicina, Instituto de Ciencias Biomédicas, Universidad de Chile, Santiago 8380453, Chile; 4 Área Técnica Farmacéutica, Facultad de Ciencias Bioquímicas y Farmacéuticas, Universidad Nacional de Rosario, Rosario S2002LRK, Argentina; jpriotti@fbioyf.unr.edu.ar; 5 Laboratorio de Nanoquímica y Química Supramolecular, Departamento de Química, Facultad de Ciencias, Universidad de Chile, Santiago 7800003, Chile; nyutroni@uchile.cl; 6 Laboratorio de Biosensores, Departamento de Química Farmacológica y Toxicológica, Facultad de Ciencias Químicas y Farmacéuticas, Universidad de Chile, Santiago 8380494, Chile

**Keywords:** cyclodextrins derivatives, drug delivery, nanomaterials, nanoparticles, polymers, controlled release, bioavailability

## Abstract

In drug delivery, one widely used way of overcoming the biopharmaceutical problems present in several active pharmaceutical ingredients, such as poor aqueous solubility, early instability, and low bioavailability, is the formation of inclusion compounds with cyclodextrins (CD). In recent years, the use of CD derivatives in combination with nanomaterials has shown to be a promising strategy for formulating new, optimized systems. The goals of this review are to give in-depth knowledge and critical appraisal of the main CD-modified or CD-based nanomaterials for drug delivery, such as lipid-based nanocarriers, natural and synthetic polymeric nanocarriers, nanosponges, graphene derivatives, mesoporous silica nanoparticles, plasmonic and magnetic nanoparticles, quantum dots and other miscellaneous systems such as nanovalves, metal-organic frameworks, Janus nanoparticles, and nanofibers. Special attention is given to nanosystems that achieve controlled drug release and increase their bioavailability during in vivo studies.

## 1. Introduction

One of the most promising and versatile tools for the construction of new drug delivery systems are cyclodextrins (CDs) and their derivatives. Their combined use with different nanomaterials has shown synergistic improvement, forming smart nanosystems with specific physicochemical properties and optimized key aspects, such as controlled release and the bioavailability of loaded drugs for different diseases.

The objectives of drug delivery research include the exploration and development of technologies that optimize the transport of pharmaceutical compounds to their target sites, in order to improve their efficacy, safety, and patient compliance. One of these methods is to modify the pharmacokinetic profile of drugs using different excipients, carriers, and medical devices, to achieve a spatio-temporal control of release, and increase bioavailability. Several strategies have been used for this purpose; CDs, and their derivatives at the supramolecular level, are one of the most prominent [1,2,3,4,5,6,7,8].

CDs are naturally obtained non-toxic cyclic oligosaccharides with a typical truncated cone shape, composed of D-glucopyranose units linked to α1-4 glycosidic bonds. The three most common types of CDs are α-, β- and γ-CDs with 6, 7, and 8 units, respectively, differing in properties like solubility, melting points, and dimensions. From a structural point of view, the hydroxyl groups are faced to the exterior, meanwhile the hydrogen and glycosidic oxygen bonds are directed towards the cone’s interior, thus giving it a hydrophilic outer surface and an internal cavity with hydrophobic character. Thus, this macromolecule acts as a host, interacting through soft bonds with molecules with a hydrophobic region and suitable dimensions, while still being soluble in an aqueous medium, a phenomenon known as the inclusion complex formation [9,10]. Despite the number of factors and different non-covalent forces involved, the host–guest complexation process, in this case the CD–drug, is simple, even when CD is part of more sophisticated molecular architectures, such as polymers, nanosponges, or nanoparticles.

Especially in the pharmaceutical field, CDs are widely and successfully used as an important tool to modulate several properties affecting the performance and therapeutic profiles of drugs. CDs enhance the apparent aqueous solubility and rate of dissolution of poorly water-soluble drugs, reducing adverse reactions such as gastrointestinal or ocular irritation and other side effects. They increase permeability through biological membranes, reduce evaporation and stabilizing flavors, and improve palatability, handling, and chemical stability in formulations [11,12,13,14,15]. Some publications report lists of CD-containing pharmaceutical formulations that have been approved by regulatory agencies in the USA, Japan, and EU countries [10,16,17]. According to the biopharmaceutical classification system, drugs can be divided into four categories based on their solubility and membrane permeability [18]. In this sense, CDs have been widely used to improve the solubility of class II and IV drugs (which have low aqueous solubility), for cancer treatments and parasitic diseases, among many other pathologies [19,20,21,22]. However, there are also reports of a complex formation for class I and III drugs, since CDs have been used to increase stability and mask flavors and odors, among other factors [23,24,25,26,27,28].

However, the limited solubility of native CDs, restrictions on the pools of possible molecules to be included, the short half-life in blood following in vivo administration, the non-control of drug release during transport, which depends on host–guest interactions and the pH and species present in the surrounding biological environment, have stalled their use as a drug carrier [29,30,31,32]. Consequently, studies into CD derivatives have advanced significantly, with chemical modifications not only improving their properties, but granting new ones. In this new stage, attention was focused on CDs modified with functional groups, among which hydroxypropyl-βCD (HPβCD), used for greater solubility and improved entrapment properties, stood out, in addition to amino-CDs and sulfo-CDs, which allow the expansion of their functions through the binding of species, by simple reactions or being grafted onto other materials.

Undoubtedly, the combination of CDs and their derivatives with nanomaterials has substantially improved the properties and applications of both, especially in the field of drug delivery [31,33,34,35,36]. Reference to nanomaterials includes all types of particles, polymeric, lipidic structures, or other types of systems that have dimensions in the nanometric scale and, therefore, can acquire new properties and characteristics. Nanomaterials have been employed to improve the biopharmaceutical properties of several drugs, through strategies such as nanoencapsulation, bioaccumulation, and passive and active targeting of the site of interest [37,38]. For example, if properly designed, nanomaterials can be applied to therapy, diagnostics, and imaging, especially for diseases associated with inflammation and tumors. In such pathological conditions, the endothelial lining of the blood vessel wall becomes more permeable than the normal state of tissue, allowing the selective accumulation of nanoparticles or nanocarriers, known as the enhanced permeability and retention (EPR) effect [35,39,40,41].

Therefore, to develop a successful drug delivery system, it is necessary to incorporate different materials that show excellent results in combination [42]. In this sense, biocompatible polymers have been an outstanding contribution. These are frequently used together with nanomaterials for pharmaceutical applications because they can improve drug loading, increase the stability and bioavailability of nanosystems, and add new functions, such as controlled release through stimuli. These advantages translate into a decrease in the frequency of doses, reducing costs and side effects. Furthermore, polymers are especially useful in non-invasive drug administration routes, such as the nasal, oral, ocular, and pulmonary routes [43,44,45].

Another relevant aspect to be considered when designing a successful drug delivery system is achieving the precise spatio-temporal control of drug release. For this, targeting agents are incorporated, which bring the system directly to the specific site of action and optimize the process. Moreover, another important aspect is the incorporation of species or materials that are stimuli-sensitive, for example, to pH, laser irradiation, or magnetic field, among others [46,47]. By generating the stimulus, it is possible to release a pharmaceutical component on demand. Currently, there are a large number of studies on smart delivery systems based on CDs, nanomaterials, or both, which incorporate targeting agents and respond to stimuli for controlled release.

Both CD derivatives and different nanomaterials are effective tools to increase the bioavailability of a pharmaceutical formulation. Therefore, its applications for different diseases are continually increasing. Oral, ocular, intranasal, or intravenous routes of administration, among others, have been explored. On one hand, CD’s contribution is emphasized in its ability to increase the solubility and permeability of the drug, as has been previously highlighted. On the other hand, nanomaterials show a more diverse contribution; for example, they are used to achieve a fast and complete, or slow and sustained absorption profile, increase aqueous solubility, stability and circulation time, or improve the arrival at the specific site of action. Therefore, CDs, derivatives and nanomaterials open new therapeutic opportunities for active pharmaceutical ingredients (API) that cannot be used effectively as conventional formulations, due to poor bioavailability or drug instability. These alternative nanosystems make it possible to develop smart drug delivery vehicles for a particular disease, even applying personalized therapy.

**Figure 1:**
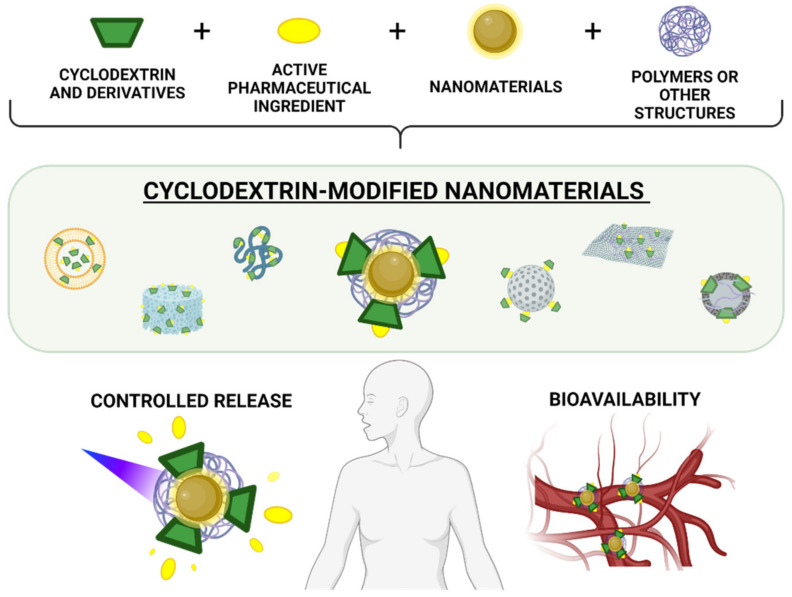
General scheme of the construction of nanosystems based on CD, polymers, and nanomaterials for their use in controlled release and enhanced bioavailability. Created with BioRender.com.

This review contains the most outstanding studies of the last five years on the design and formulation of nanomaterials modified with CDs and their derivatives for drug delivery, and discusses their advantages and disadvantages for different diseases. A classification of the main types of nanosystems is addressed, and their composition, strategies for controlled drug release, and the latest advances in bioavailability are discussed in depth (Figure 1).

## 2. Classification of Cyclodextrin-Modified Nanomaterials

**Figure 2:**
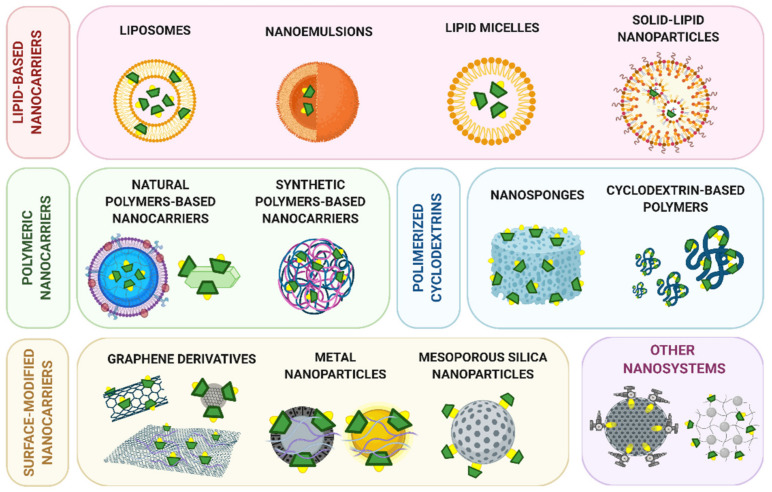
General classification of CD-modified nanomaterials applied to drug delivery composed of CDs and their derivatives, drugs, polymers, and nanomaterials, considering the characteristics and composition. Created with BioRender.com.

Several classifications of CD-modified nanomaterials were described depending on nanosystem characteristics and composition [31,34,48]. The following subsections discuss the main nanosystems that incorporate CD derivatives (Figure 2), classified by the chemical nature of the nanomaterial, and focusing on the most relevant findings described in the literature of the last five years.

### 2.1. Lipid-Based Nanocarriers

Lipid-based nanocarriers are perhaps the most popular nanomaterials used in combination with modified CDs, being at the forefront of developing new systems for drug delivery. Biocompatible and biodegradable components form these systems; they are versatile, and offer advantages related to controlled release, stability, and drug loading. Lipid-based nanocarriers can be used for targeted delivery, can be administered by different routes, and can even co-load both lipophilic and hydrophilic drugs [49]. Moreover, they have shown improvements in different drugs’ pharmacokinetics, efficacy, and safety [50,51]. However, some considerations should be taken regarding the loading capacity of these systems and the passive release that can be observed mainly in systems designed to transport hydrophilic molecules [52]. Relevant studies on the lipid-based nanosystems incorporating CD derivatives are presented below, and a summary is presented in Table 1.

#### 2.1.1. Liposomes

Liposomes are small artificial vesicles of spherical shape, consisting of one or more lipid bilayers, mainly composed of amphipathic phospholipids with a non-polar tail and polar head, enclosing an aqueous interior space [53,54]. Different strategies have been developed to combine liposomes and CDs, for example, the inclusion of complexes within the aqueous nucleus of different liposomes, a strategy called “drug-in CD-in liposome systems”. Russo Spena et al. applied this strategy for the development of a new therapy for high-grade serous ovarian cancer by the delivery of a Pin1 inhibitor encapsulated in modified CD and remotely loaded into pegylated liposomes [55]. This liposomal formulation accumulates preferentially in the tumor, and has a desirable pharmacokinetic profile. The liposomes were able to alter Pin1 cancer-driving pathways through the induction of a proteasome-dependent degradation of Pin1 and were found to be effective in curbing ovarian tumor growth in vivo.

However, the ability of single-loaded liposomes to encapsulate drugs or inclusion complexes in this nucleus was limited. Due to this fact, some authors also incorporated complexes in the bilayer of the liposomes, obtaining systems with inclusion complexes in the nucleus and the nanomaterial shell. These materials are known as “double-loaded liposomes” [56]. In 2018, Bhatt et al. enhanced the loading efficiency and pharmacokinetics of paclitaxel using this combination strategy [57]. The simultaneous incorporation of drugs in the liposomal core by pre-encapsulation in dimethylβCD, along with incorporation in the bilayer, presents a promising strategy for improving loading efficiency and stability issues. The designed double-loaded PEGylated liposomes were superior in performance to Taxol^®^, with a prolonged release profile, low hemolytic potential on red blood cells, and higher cytotoxicity and antiproliferative activity on the cancer cell line. Pharmacokinetic advantage was evidenced by the longer circulation time and increased plasma concentration in rats, and a lower toxicity potential tested in mice.

Deformable liposomes have also been developed to improve their effectiveness as drug carriers, due to their high flexibility and adaptability to improved penetration through biological membranes. These systems can squeeze intact among the *stratum corneum* cells and then reach the deep skin layers. Recently, Mura et al. improved Butamben anesthetic efficacy by the development of deformable liposomes bearing the drug as a CD complex [58]. Deformable double-loaded liposomes, bearing the lipophilic drug in the bilayer and the hydrophilic βCD and methyl-βCD complex in the aqueous core, were compared with deformable single-loaded liposomes. All vesicles showed homogeneous dimensions (i.e., below 300 nm), high deformability, excellent entrapment efficiency, and allowed a more controlled and prolonged drug release during in vivo experiments. In vivo studies on rabbits proved that double-loaded liposome formulations significantly increased the intensity and duration of the Butamben anesthetic action, owing to the presence of the drug–CD complex in the vesicle core, acting as a reservoir.

Finally, the nanoliposomes (NLs), which are integral parts of cell plasma, have been identified as potential biocompatible drug carriers, enhancing the bioactive agents’ performance by improving their in vitro solubility and bioavailability. In addition, due to their specific and unique physicochemical and biological properties, and their easy size manipulation, the successful application of NLs as drug carriers will play a vital role in pharmaceutical applications [59]. Recently, Souri et al. loaded β-CD/vitamin E inclusion complex in NLs and coated this nanocarrier with sodium caseinate. The coated LPs exhibited a better-sustained release profile than the uncoated NLs in the simulated gastrointestinal condition, and the systems showed promissory advantages and potential applications in the nutraceutical and medical fields [60].

#### 2.1.2. Nanoemulsions

Nanoemulsions are transparent oil-in-water or water-in-oil systems. Their droplets cover a size range between 50 and 200 nm, and they are characterized by excellent stability in suspension due to their very small size and significant steric stabilization between droplets. Likewise, different lipid nanocarriers have been developed regarding nanoemulsions as a template [50,61]. Recently, Hou et al. designed a nanoemulsion of cinnamon essential oil, co-emulsified with HPβCD and Tween-80 to protect the ingredients and sustain the release, to exert long-term antibacterial effects [62]. The results showed that the hydrophobic cavity could include essential oils and form inclusion complexes to inhibit aggregation and reduce the particle size of the nanoemulsion. The system showed good antibacterial effects against *Escherichia coli* and *Staphylococcus aureus*. The release time, the cumulative release amount, and thermal and time stability indicated that the addition of HPβCD reduced the loss of essential oil, decreased the release rate, and contributed to storage stability.

Additionally, more complex strategies, such as multiple nanoemulsions, can be applied in combination with CD. In 2018, Pangeni et al. improved oral absorption of pemetrexe by forming a complex with a permeation enhancer with HPβCD and poloxamer 188, which was incorporated into water-in-oil-in-water nanoemulsions in a supersaturated state [63]. After the complex formation, the drug showed similar cytotoxic and inhibitory effects on cancer cell proliferation/migration. Furthermore, the nanoemulsion synergistically improved the intestinal membrane permeability and epithelial cell uptake. The oral bioavailability of loaded nanoemulsion in rats was 223% higher than the oral pemetrexe, and significantly suppressed tumor growth in Lewis lung carcinoma cell-bearing mice. These results imply that loaded nanoemulsion is an effective and promising delivery system for enhancing oral absorption.

#### 2.1.3. Solid-Lipid Nanoparticles and Nanostructured Lipid Carriers

Solid-lipid nanoparticles (SLNs) are colloidal systems that present sizes from 50 to 1000 nm, depending on the process and conditions used in their preparation. They offer similar advantages to liposomes and nanoemulsions, but are more effective for the drug protection of chemical degradation and drug control release [53]. SLNs consist of a solid lipid core with a monolayer coating of surfactants. Their hydrophobic lipid core may contain fatty acids, saturated fatty acids (e.g., stearic acid), triglycerides (e.g., tristearin), glyceride mixtures (e.g., Imwitor), or waxes (e.g., cetyl palmitate). They are solid at both room temperature and human body temperature, and drugs may be dissolved or dispersed in them [64,65,66]. The main drawbacks of these formulations are associated with drug expulsion during storage and undesired particle growth by agglomeration or coagulation, affecting the stability of the nanosystems and generating possible problems in the administration of the dose.

Recently, a layer-by-layer assembly of core-corona structured SLN with βCD polymers was developed by Amasya et al. [67]. A negatively charged SLN core was successfully coated by positively charged polyβCD-N+ to obtain 1-Layer SLN and 2-Layers SLN. These nanoparticles were produced by the sequential deposition of oppositely charged polyβCDN+ and polyβCDS− using the layer-by-layer technique. The presence of the oppositely charged polymer layers was found by the alteration of surface charge from negative to positive values, and an increased density was achieved compared with the core SLN. The results also revealed that the drug release was mainly controlled by diffusion, and that polyβCD could enhance the slow/controlled release of drugs. Cytotoxicity assay suggested that the novel, hierarchical core-corona structured SLNs did not have cytotoxic effects on healthy cells, and could be safely used as drug carriers.

On the other hand, nanostructured lipid carriers (NLC) have been developed as the second generation of SLN, to increase drug loading and prevent drug escape [68,69]. In contrast to the SLN, they present a less organized solid-lipid matrix, that results from a blend of solid lipids and liquid lipids (oils) [70,71]. NLC presents high encapsulation efficiency, stability, and suitability for industrial processing, and a slow-release [72]. In 2018, Cirri et al. investigated the combination of CD complexation and NLC, to obtain a liquid oral pediatric formulation of hydrochlorothiazide, endowed with safety, dosage accuracy, good stability, and therapeutic efficacy [73]. The presence of the HPβCD complex in NLC increased the entrapment efficiency and stability of the nanosystems and allowed a complete controlled drug release from the formulations. The NLC formulation showed a significantly more intense and prolonged diuretic effect of the drug during in vivo rats studies, proving the actual successfulness of the proposed approach. 

Solid inclusion complexes with CDs were also used to overcome the volatility and solubility problems of essential oils of pharmacological interest. However, they lack the many dermatological advantages of lipid nanoparticles. Pires et al. evaluated the encapsulation of the HPβCD inclusion complexes of *Lippia origanoides* essential oil, Thymol, on NLC to follicular accumulation and controlled delivery [74]. Whereas CD effectively overcame the volatility and low aqueous solubility of APIs, NLC itself controlled the drug release, enabling thymol penetration into the skin. The combined approach resulted in a synergistic effect of 20% drug released after 12 h, with a shelf life of 6 days. The results suggest the developed system maintained all skin interaction aspects of lipid colloids, including follicular accumulation forming a depot for controlled delivery.

#### 2.1.4. Lipid Micelles

Lipid micelles are colloidal dispersions that have a hydrophobic core and a hydrophilic shell. They are formed spontaneously, from amphiphilic molecules or surfactant agents at specific concentrations and temperatures [49]. In a recent study, an astaxanthin HPβCD inclusion complex that self-assembled into micelles in an aqueous solution was prepared, and achieved solid-phase loading of the drug by Su et al. [75]. The authors improved the aggregation characteristics of the micelles by adding a small flexible substance, glyceryl monostearate, improving the solubility and antioxidant activity of the drug. The results showed that the formation of the inclusion complex increased free radical scavenging activity and the solubility of the API. The pharmacokinetic studies showed that the drug bioavailability increased four-fold, and the tissue distribution experiments showed that the system targeted the liver to exert its antioxidant effects. This work illustrates an unprecedented preparation method by mechanical ball milling, and represents a significant innovation in the formation of self-assembled micelles.

### 2.2. Polymeric Nanocarriers

Due to their structural features and versatility, polymers can be helpful to increase drug bioavailability, control release, or target drug delivery. There are many available biocompatible polymers that can be modified for almost any pharmaceutical purpose. The following is a summary of the last scientific literature involving polymers, natural or synthetic, combined with CDs or chemically modified with CDs for pharmaceutical nanoformulations. A summary of the most important data can be found in Table 1.

#### 2.2.1. Natural Polymer-Based

The natural polymers found in nanoparticles in combination with CDs include cellulose, chitosan, and hyaluronic acid. Cellulose is the most abundant natural polymer. Commercial cellulose derivatives are widely used and extremely valuable in the pharmaceutical industry, playing roles such as coatings for control release, and enhancing the solubility and bioavailability of poorly soluble drugs [76,77].

The synthesis of nanocrystals composed of cellulose loaded with βCD has been studied and has shown promising results. In 2016, Ndong-Ntoutoume and collaborators reported the complexation of cellulose nanocrystals which present negative charges on the surface, with βCD functionalized with glycidyltrimethyl ammonium chloride (a cationic CD) [78]. Authors found that curcumin-CD/cellulose nanocrystals were three to four times more effective than curcumin alone, or curcumin-CD complex (without cellulose), demonstrating that the natural polymer cellulose provided an increase in the cellular uptake. In a subsequent work of the same research group, Caillaud and co-workers have shown an exhaustive in vitro and in vivo evaluation and reported the improvement of curcumin pharmacokinetics and Charcot-Marie-Tooth 1A phenotype in transgenic rats given curcumin-CD/cellulose nanocrystals [79]. This nanosystem reached a higher bioavailability, even at a dose 250 times lower than that of free curcumin, meaning nanocrystals have considerably improved curcumin pharmacokinetics. Additionally, the nanocrystals showed a prolonged release of curcumin in plasma for at least 12 h after intraperitoneal injection, whereas curcumin alone was not detectable after 8 h. These findings make curcumin attractive for the treatment of peripheral neuropathies.

Chitosan is a biocompatible and biodegradable polymer, widely used in nanoparticles for drug delivery [1]. Chitosan is obtained from partially deacetylated chitin extracted from crab and shrimp shell waste, making it an environmentally sustainable biomaterial. In its chemical structure, chitosan has groups that can be modified to obtain a polymer with different applications and different administration routes [80,81]. An interesting approach within pharmaceutical nanosystems based on CDs and chitosan relies on the crosslinking between chitosan (positively charged) and tripolyphosphate (negatively charged). In these nanoparticles, chitosan functions as a polymer for the sustained release of the drug, included in a nonionic CD and immersed in a polymeric network. Tang and coworkers incorporated salazosulfapyridine, a drug for the treatment of rheumatoid and inflammatory bowel diseases, in a CD inclusion complex with dimethyl-βCD that showed a significant increase in drug loading efficiency [82].

By changing the chemical composition, nanogels can be designed for drug co-delivery and controlled release at different pH values. Song et al. designed nanogels based on chitosan derivatives and HPβCD, coated with red blood cell membrane, that co-encapsulated a hydrophobic drug, paclitaxel, and a biomolecule, interleukin 2 (IL-2), to achieve the synergistic antitumor effects of chemotherapy and immunotherapy [83]. HPβCD-acrylate was introduced into the nanogel to enhance paclitaxel entrapment and control its release. Then, the erythrocyte membrane was introduced to protect IL-2 and extend the circulation time in the blood. The membrane may also promote binding to IL-2 receptor. This smart nanodevice exhibits controlled release and a rapid response to pH decrease, which is useful to targeted drug delivery in the tumor microenvironment. It also has a high loading capacity for paclitaxel, extends IL-2 half-life, and enhances drug penetration in the tumor, as demonstrated by in vitro studies. Researchers demonstrated an increase in the pharmacokinetics, distribution, and improved antitumor efficacy of the nanosystem on in vivo studies.

Hyaluronic acid is a linear anionic biopolymer found in the connective, epithelial, and neural tissues. Hyaluronic acid contains functional groups that can be functionalized for stimuli-response. When nanosystems are coated with hyaluronic acid, the surface negative charge prevents the clearance by the reticuloendothelial system [84]. Biocompatible and enzymatic biodegradable nanoparticles for cancer therapy were developed by Yang et al. in 2016 [85]. Such nanoparticles were designed with a hydrophobic core hosting called PorTaxol, porphyrin modified paclitaxel, a fluorescent prodrug, and a hydrophilic polymeric shell consisting of hyaluronic acid, modified with permethyl-βCD. Hyaluronic acid provided a system for targeting cancer cells, as it binds to its receptor and promotes internalization by endocytosis. Although studies in vitro showed these nanoparticles had similar antiproliferative activity to Taxol against cancer cells, the nanocarriers exhibited lower side effects than the commercial drug.

#### 2.2.2. Synthetic Polymer-Based

The field of synthetic materials for biomedical applications has experienced fast growth in the last three decades. Among these, synthetic polymers play a leading role, mainly due to the tremendous amount of available marketed monomers and the feasibility of functionalization. Indeed, it is possible to synthesize biocompatible polymers with the desired properties [86,87]. For example, synthetic and commercially available polymers, such as polyvinyl alcohol and Pluronics^®^, have been studied by Dash and coworkers as stabilizers to increase the entrapment efficiency in curcumin nanoparticles encapsulated in HPβCD from 1–5% (no polymer) to about 60% (with polymer) [88]. The presence of the polymer restricts the aggregation of curcumin that leaves the CD cavity upon solvent evaporation. Finally, authors have shown that curcumin nanoparticles combined with PEGylated liposomal doxorubicin (DOX) might be useful for a co-treatment in cancer therapy to reverse DOX resistance.

On the other hand, to increase econazole dissolution and bioavailability for ocular administration as an antifungal treatment against *Candida albicans* and tinea, nanoparticle suspensions (or simply nanosuspensions) were prepared by nanospray drying, using CDs and polymers by Maged and collaborators [89]. Two commercial βCD derivatives were combined with different synthetic stabilizer polymers, and compared through in vitro studies. The combination HPβCD and Tween 80 was shown to be the best in increasing drug release and preventing econazole nitrate aggregation during storage for one year, and was selected for bioavailability studies in rabbits. The addition of chitosan to the nanosuspension containing HPβCD and Tween 80 not only preserved the stability for one year but also increased econazole bioavailability in tears, due to an increase in the wettability and viscosity of chitosan, and its positive charge.

The two most common strategies to modify synthetic polymers with CD are covalent and supramolecular binding. One example of the first is the nanosystem recently developed by Han et al., where CDs were linked to a polymeric chain for drug delivery [90]. In this study, PEG derivate was synthesized as the polymer backbone, and different amino-βCDs were grafted. The loading properties were tested with pyrene to encapsulate ferulic acid as API. The final system could self-assemble into core-shell structured nano-micelles with a negative surface and high drug-loading capacity. Moreover, pH-triggering behavior demonstrated an increase in drug release under a weak acid condition in an in vitro release study. After intravenous injection, nano-micelles accumulated in the rat liver, indicating a potential application in cancer therapy and/or liver-associated diseases.

Moreover, an example of the second strategy involves supramolecular assemblies to design nanosystems or micelles for the drug delivery of DOX. This approach was developed by Zhang and coworkers [91]. The system was built through the host−guest interactions between a host polymer, βCD graft to poly(2-(dimethylamine)ethyl methacrylate), and a guest polymer, azobenzene-modified poly(ε-caprolactone), encapsulating the drug inside the package. These assemblies or micelles showed controlled release and similar or superior antitumor activity, compared with free DOX. Supramolecular polymers, in this case, showed a response to pH and light (Azobenzene) as internal and external stimuli. Polymers assembling in supramolecular micelles can be chosen to respond to different intracellular environmental stimuli (e.g., temperature, redox potential, enzymes, glucose), or external environmental stimuli (e.g., light, electricity, magnetism, ultrasound). All this aims to improve the availability of drugs in specific tissues and minimize side effects.

An interesting approach in the study of intelligent drug delivery systems is the use of stimuli-responsive polymers. Among them, poly-*N*-isopropylacrylamide is a temperature-responsive polymer that aggregates reversibly, and can be dispersed onto nanoparticles. In 2019, Das et al. developed nanoconjugates based on a copolymer of βCD-Maleic anhydride and poly-*N*-isopropylacrylamide, iron oxide nanoparticles, fluorescein, and folic acid for multiple functions [92]. The thermal response of the polymer was intended to target cancer cells, due to the temperature difference between normal cells (37 °C) and cancerous cells (43 °C). The loading of two therapeutic agents on the nanosystem was studied: DOX and curcumin. The system allowed: pH- and temperature-response drug release, as a contribution of CD and polymer; movement tracking in the body and cellular uptake monitoring, due to the characteristics of iron oxide nanoparticles and fluorescein; targeted delivery and therapy using polymer, folic acid, curcumin, and DOX. The in vivo studies corroborated the in vitro data, showing a significant tumor regression when drug-loaded nanoconjugates were injected into the animals. In this way, the synthesized nanoconjugates showed a potent application to act as effective theranostic agents (integration of therapy and diagnosis) in the field of cancer therapy.

**Table 1:** Summary of the most important data based on the classification according to the type of nanosystem, type of CD used, types of polymers, and results regarding drug loading, release mechanism, and in vivo studies (if available) from Section 2.1 and Section 2.2.

	System	CD	API	Loading Capacity	Loading Efficiency	Drug Release Mechanism	In Vivo Studies	Ref.
Lipid-based nanocarriers derivates	CD–drug loaded liposomes	Amino-deoxy-βCD	Pin1 inhibitor	-	91%	Slow diffusive release	Pharmacokinetics, biodistribution, and efficacy	[55]
	Double-loaded liposomes	Dimethyl-βCD	Paclitaxel	1.2 mg/mL	93%	Slow diffusive release	Pharmacokinetics and acute toxicity after intravenous administration	[57]
	Single and double-loaded deformable liposomes	HPβCD, SBEβCD and MEβCD	Butamben	0.01%	92–100%	Diffusive release	Ex vivo permeation and in vivo anesthetic effect	[58]
	Nanoemulsion with HPβCD and Tween-80	HPβCD	Cinnamon essential oil	-	-	Slow diffusive release	-	[62]
	Multiple nanoemulsion (w/o/w) with HPβCD and poloxamer 188	HPβCD	Pemetrexed	-	95%	Quick diffusive release	Oral bioavailability and in vivo tumor growth inhibition effect	[63]
	SLN capped with βCD polymers	βCD	Benzophenone	9–12%	72–96%	Higuchi and Korsmeyer–Peppas kinetics	-	[67]
	NLC loaded with CD–drug complex	HPβCD and SBEβCD	Hydrochlorothiazide	2–4%	40–88%	Quick diffusive release	Diuretic activity after oral administration	[69]
	NLC loaded with CD–drug complex	HPβCD	Thymol	2.2%	79%	Higuchi kinetic	Ex vivo skin permeation	[74]
	Micelles assembled from HPβCD and glyceryl monostearate	HPβCD	Astaxanthin	2.7%	100%	pH change	Oral bioavailability, tissue distribution	[75]
Polymeric nanocarriers	CD-cellulose nanocrystals	Glycidyltrimethyl ammonium chloride-βCD	Curcumin	91 mg/g	9%	More likely, cell internalization due to endocytosis followed by release into lysosomes	Bioavailability, in vivo nervous function	[78,79]
	CD–drug inclusion complex loaded chitosan nanoparticles	Dimethyl-βCD	Salazosulfapyridine	3–10%	80–90%	Degradation of polymeric matrix	-	[82]
	Red blood membrane-coated nanogels formulated	HPβCD acrylate	Paclitaxel and IL-2	93% (500 µg Paclitaxel)	32% (500 µg Paclitaxel)	pH change	Drug release in tumor microenvironment, bioavailability, biodistribution, antitumor efficacy, immune response	[83]
	Polysaccharide-based noncovalent assembly for targeted drug delivery	Permethyl-β-CD	Porphyrin modified paclitaxel	31%	85%	Enzyme-triggered drug release	-	[85]
	Nanoformulation based on PEGylated liposomal and nanocurcumin	HPβCD (+citric acid)	DOX + Curcumin	-	>95% (data not shown)	-	-	[88]
	Ocular nanosuspension based on commercial polymers	Methyl-βCD/HPβCD	Econazole Nitrate	43–52%	-	Degradation of polymeric matrix	Ocular irritation, Bioavailability in tears	[89]
	Amino-βCD-containing polymers nanoassemblies	Amino-βCD with various alkyl chains	Ferulic acid	4%	-	pH change	Biodistribution	[90]
	Dual stimuli-responsive supramolecular self-assemblies	βCD-graft-poly(2-(dimethylamino)ethyl methacrylate)	DOX	13%	66%	pH change and UV irradiation responsive release	-	[91]
	Multifunctional nanoconjugates	βCD-Maleic anhydride	Curcumin and DOX	0.45 g/g and 0.32 g/g	88%	pH change and temperature change	Blood markers, gene expression in liver tissue	[92]

### 2.3. Polymeric Nanosystems Based on Cyclodextrins

The first covalent CD-based polymer networks for pharmaceutical purposes were prepared in the 1980s [29,93]. These were composed of CDs, which constituted the unit of the system, and a crosslinking agent, commonly called nanosponges if they were of nanometer size. In the last few years, the most commonly used unit has been the βCD, whereas the most common crosslinking agents have been epichlorohydrin and diphenyl carbonate or similar species. Until now, a wide variety of polymeric CD derivatives with multiple applications have been studied for drug delivery, imaging, and theranostics [13,36,94]. The main objective of their use is to increase the solubility of poorly water-soluble drugs, and increase their bioavailability in pharmaceutical formulations through an improvement in their release profile [35,95,96]. Table 2 presents a summary of the most recent works on polymeric nanosystems based on CDs.

An advantage of working with epichlorohydrin CD-based polymers is their commercial availability in companies like Cyclolab^®^. For example, Yakavets and col. have loaded temoporfin (Foscan^®^) on commercial βCD and carboxymethyl-βCD nanosponges, reporting a higher association constant in both cases than the monomer [97]. The main goal was to study the accumulation and penetration into 3D tumor spheroids, an innovative model to evaluate therapy for cancer. Authors demonstrated that the nanosystems had a slower release profile than the native βCD. Furthermore, if the concentration was properly modulated, temoporfin diffusion occurred without interacting with the perimeter cells in the smoother penetration profile and the homogeneous distribution of the drug in all cells of the spheroid. Similarly, Giglio and cols. compared the loading of sorafenib (Nexavar^®^) on a commercial βCD-based polymer and a synthesized βCD-based oligomer, observing two times higher affinity for oligomers with up to 80 μM of drug [98]. The loading systems showed effectiveness in triggering apoptosis in cancer cell lines, low overall toxicity in vivo, and almost no toxicity in the liver compared with the drug on its own.

Even these systems can be functionalized with specific vectorization agents through covalent or supramolecular strategies. For example, Viale et al. studied the loading of DOX into commercial polymers and oligomers synthesized from βCD, and their covalent functionalization with the RGD peptide for tumor cell targeting [99]. Both systems allowed an increase in cytotoxicity and the anti-proliferative capacity of DOX on cancer cell lines. In addition, the functionalized oligomer nanosystems for targeting showed a two-fold increase in cytotoxicity. On the other hand, Cordaro and col. constructed a fluorescently labeled commercial poly-βCD-based nanoassembly loaded with the drug diclofenac, to treat inflammation in osteoarticular diseases and a probe as a potential theranostic [100]. Probe loading did not disturb drug loading, leading to a loading capacity of 1% and a loading efficiency of 92% for diclofenac. A slow-release profile was observed, with no traces of the probe, which, together with its physicochemical properties, makes it a promising candidate for syringeable formulations. Furthermore, no detrimental effects on proliferation and viability, and rapid cell clearance were observed.

Synergistic therapy, or co-loading of therapeutic or diagnostic agents, is another remarkable advantage of these systems. The co-loading of Ethionamide and pharmacological booster onto synthesized polymeric βCD nanoparticles was studied by Prof. Greg’s group [101,102]. Notably, the polymers increased drug solubility 10-fold over βCD, and the booster did not compete for inclusion. In addition, the loaded drug formulation successfully traversed a MicroSprayer membrane that simulated the pulmonary route. In a second publication, the efficacy of the drug formulation in cells infected with *M. tuberculosis* was evaluated. In addition, in vivo studies showed increased efficacy of the system in mouse lungs over no-drug activity alone, without enhancers or nanocarriers, showing the potential for the treatment of tuberculosis.

However, the study of CD-based polymeric derivatives is not only limited to the use of epichlorohydrin; new derivatives using novel crosslinking agents have also been investigated. An interesting synthesis using diglycidylether as a crosslinker and HPβCD was investigated by Thatiparti and collaborators [103]. Polymers made with crosslinkers of different chain lengths showed better mechanical and physicochemical properties, such as improved stability concerning other similar systems, as well as a different loading capacity. The inclusion of the drugs novobiocin and vancomycin was studied in these pseudopolyrotaxane CDs derivatives. The best loading capacity was demonstrated for the polymers with shorter chain crosslinkers, with values of around 25 ± 1% and 8 ± 1%, respectively. Both drugs showed a slower release profile in CD-based carrier systems than in non-specific polymers, in addition to improved antibacterial activity and cell adhesion.

For its part, research on nanosponges applied to drug transport has significantly advanced in recent years. Generally synthesized based on diphenyl-carbonate and similar materials, they make it possible to improve CD’s supramolecular properties and expand the strategies that can be used for their application. For example, Moin and co-workers have reported the simultaneous loading of three drugs: paracetamol, aceclofenac, and caffeine into diphenyl carbonate-based nanosponges for the potential therapy and acute treatment of chronic-pain-related ailments [104]. With a size of 200 nm and a loading efficiency of approximately 90% for the best optimization, it was possible to directly compress the nanosystem into tablets to study its dissolution and release profile. The tablet showed a slow and sustained release profile, which is promising for use in combination therapy. On the other hand, diphenyl carbonate-based nanosponges have been used to load two therapeutic agents of different natures simultaneously: drugs and gold nanoparticles.

Remarkably, the use of a nanocarrier for these dimensions not only increases the amount of drug available in a formulation, by increasing its solubility and improving its stability due to supramolecular inclusion, but also reduces the time of elimination from the bloodstream and thus increases the bioavailability of the drug. This has been studied by authors in recent years through in vivo bioavailability assays, with favorable results. For example, in 2017, Shringirishi and co-workers studied the bioavailability and release profile of nifedipine loaded in βCD-based nanosponges, aiming to improve the treatment of angina pectoris and hypertension [105]. The loading efficiency shown by the nanosystem was 78%, and the size was 430 nm. Drug release was studied in simulated gastric fluid exhibiting a burst release for the first four hours, then switched to a controlled delivery for the subsequent 24 h. Its evaluation on in vivo models showed an oral bioavailability 3.6 times better than the control formulation, and an adequate stability for 6 months. In 2018, Zidan et al. evaluated the release of atorvastatin calcium loaded on CD-based nanosponges, their oral bioavailability, and their therapeutic effect on reducing cholesterol and triglycerides levels [106]. The polymers were synthesized using carbonyldiimidazole in different ratios. The maximum drug-loading capacity on the sponges was 34%, with a size of 420 nm. The in vitro release study also indicated a biphasic pattern, with a rapid release of 58% after 1 h, and then an extended release of 6 h, which was better than the dissolution of only 43% of the drug alone. Studies on in vivo models have indicated an improvement of pharmacokinetic and pharmacodynamic parameters, increasing the bioavailability of the drug 2.13 times, and significantly improving therapeutic efficacy in animals with fatty livers. In 2019, Amin and collaborators studied the bioavailability and release profile of Febuxostat loaded on βCD-based nanosponges for the treatment of gout [107]. The loading efficiency ranged from 88 to 100% for nanosponges with different cross-linking agent (diphenyl carbonate) ratios, and sizes that ranged from approximately 220 to 300 nm. The nanosponge-based tablets loaded with the drug showed adequate properties, with a controlled biphasic release profile, allowing 30% release at the first hour followed by controlled release close to 75% after 6 h. Its evaluation on in vivo models showed a better bioavailability than the commercial formulation in all pharmaceutical parameters, and a relative bioavailability of 217%.

Thus, CD-based covalent polymerics have been widely applied in the last 5 years due to the change in their physicochemical properties, such as increased solubility, stability, and desirable mechanical properties in pharmaceutical formulations such as tablets. In addition, crosslinking allows the formation of inclusion and non-inclusion compounds, increasing the maximum amount of drug possible to be transported, making them more efficient hosts [108]. In addition, the ability to increase drug bioavailability has been proven in studies on in vivo models. However, the release of the drug from the formulation is always tested in vitro, in biological media or cells, through a gradient. Delving into this point, by means of in vivo studies, should be a priority, especially due to the high constants of stability CD–guest commonly reported that would make it difficult to release at a specific site. An interesting strategy proposes the interaction of these systems with other therapeutic agents to control the release of stimuli [2,3,4,109]. An example of this is the work done by Asela et al. [110]. They report the loading of phenylethylamine and 2-amino-4-(4-chlorophenyl)-thiazole on nanosponges with a loading capacity of 90% and 150%, respectively; this resulted in an eight-fold increase in the amount of drugs that can be carried by these nanosystems. In addition, the exposure of the drug functional groups allows the simultaneous loading of gold nanospheres at an efficiency of 85%, as a second therapeutic agent in each nanosystem. This strategy could turn nanosystems into potential stimuli-responsive smart systems for controlled drug release, for use as antidepressants and antimicrobials systems, respectively [2,3,111].

### 2.4. Graphene Derivatives

Graphene and its derivatives are versatile materials in the biomedical field; for example, they are applied for drug delivery, photodynamic therapy, imaging, or theranostic uses. In drug delivery, the systems that incorporate graphene-derived nanomaterials mainly seek to increase the loading capacity of drugs and control their release by applying stimuli such as alternating the magnetic field (AMF), or changes in pH or temperature [112,113]. In this field, there are various studies on polymers and CDs along with graphene derivatives, such as fullerenes [114], reduced graphene oxide [115], or carbon nanotubes [116]; however, the leading works are those that incorporate graphene oxide (GO) [117].

The work of Das et al. stands out for the development of a multi-walled carbon nanotube-based nanosystem, modified with βCD and isopropylacrylamide polymer, which was functionalized with fluorescein and folic acid, and co-loaded with curcumin and DOX [116]. The loading efficiency was 92%. The highest release percentages were recorded at temperatures of 40 °C, and there were different pH values for each drug. In an acidic environment (pH 5.0) 94% curcumin was released, and in a basic environment (pH 7.4), 96% DOX was released. Similar release percentages were obtained when these systems were irradiated with a laser (at 808 nm) that induced the photothermal effect: 95% for curcumin (laser + pH 5.0) and 98% for DOX (laser + pH 7.4). In in vitro studies performed for these drug-loaded nanosystems, a mortality rate was obtained in HeLa and MCF-7 cells of 70%, which reached 80% after irradiation, consistent with the release results. During in vivo studies performed on cancer-induced BALB/c mice, a notable decrease in the levels of biomarkers MMP-9 and AFP was demonstrated, indicating tumor regression, which was also evident in the histoarchitecture of the liver. Finally, this showed that the combined use of these materials improves therapeutic efficacy in cancer.

Regarding the development of GO-based nanosystems, in 2018, Borandeh and col. synthesized a CD-modified GO-based nanocarrier for potential therapy in cancer, using L-phenylalanine as a linker [118]. Notably, the nanosystem’s DOX loading efficiency and loading capacity were 78.7% and 85%, respectively, being nearly twice as high as the corresponding value of GO and GO-phenylalanine. Furthermore, a study of DOX release from the nanocarrier was performed at values of pH 7.4 (the physiological pH) and 5.3 (the pH of cancer cells). In 72 h, only 12% of DOX was slowly released at a basic pH, whereas at an acidic pH, a release of 40% was achieved. Regarding cell viability studies, GO-phenylalanine-βCD nanocarrier had no noticeable toxicity (on MCF-7 cells), whereas the nanocarrier loaded with DOX had an outstanding killing capability. The system designed by Pooresmaeil and Namazi was based on graphene oxide, and magnetic nanoparticles of Fe_3_O_4,_ and Mono-6-EDA-βCD covalently linked, for an anticancer effect [119]. Loading efficiency and loading capacity were evaluated for two drugs: 37% and 3.7% for DOX, respectively, and 23% and 2%, for methotrexate (MTX), respectively. Additionally, its release from the nanocarrier was demonstrated after 24 h at acidic pH (5.0), 34% for DOX and 19% for MTX. 

Siriviriyanun and col. used graphene oxide nanosheets, covalently modified with amino-βCD and poly(amido amine) dendrimers, forming the GO-CD-DEN system [120]. The high loading capacity of DOX in graphene oxide was improved by the presence of the CD, at 1.2 times higher; however, it decreased with the presence of the polymer. The release of the drugs DOX, camptothecin, and protoporphyrin IX (photosensitizer) from the GO-CD-DEN system was demonstrated at the same pH values. DOX release at pH 5.5 was higher than at pH 7.4, indicating its release was Ph-responsive. On the contrary, camptothecin release at pH 7.4 was greater than at pH 5.5. Protoporphyrin IX was released in an amount less than 10%, although the low percentage of release from GO-CD-DEN allowed for improved photocytotoxicity towards cancer cells (MDA-MB-231 and HeLa cells), compared with free protoporphyrin IX.

On the other hand, Wang and col. designed a new supramolecular hydrogel, composed of graphene oxide and αCD [121]. In addition, the system contained poly(*N,N*-dimethylaminoethyl methacrylate) and poly(ethyleneglycol) monomethyl ether polymer. With this, the supramolecular hydrogel can increase drug loading through host–guest interactions and respond to NIR light, temperature, and pH, releasing the anticancer drug 5-fluorouracil. Under the conditions studied, the maximum amount of drug released was close to 80%, when the temperature was increased to 55 °C for 20 min after 4 h at 37 °C. Using NIR irradiation, the controlled release of 5-fluorouracil was close to 70%, and occurred after 4 h when the system was irradiated for 20 min. The cumulative releases for 48 h of 5-fluorouracil at pH values of 9.4, 7.4, and 5.4 at 37 °C were 64%, 73%, and 95%, respectively, indicating that the release increased with the decrease of pH. Therefore, graphene oxide sheets were used as a core material for additional crosslinking, conferring thermal stability. Additionally, graphene oxide absorbed NIR light, transforming a portion into local heat to trigger the gel−sol transition. 

Zhang and col. performed a different system from those previously mentioned [122]. Their work combined graphene oxide, Ni magnetic nanoparticles, a βCD derivative, and peptide-grafted hyaluronic acid that acted as a vector for the nanocarrier. The nanosupramolecular system can control DOX release by applying an AMF. The presence of multiple components increased the loading capacity in the system, based on magnetic graphene oxide with CD being at ~18%, and the system with peptide and hyaluronic acid being at >36%. Furthermore, a study in tumor cells showed a greater efficacy in releasing the drug DOX under AMF stimulus when the peptide with hyaluronic acid was included to the supramolecular system. Finally, it is important to mention that the complete nanosystem can target the mitochondria, effectively releasing DOX to both the mitochondria and the nuclei and causing the death of tumor cells, which was aggravated by the application of AFM.

Recently, Sattari and col. designed a novel system composed of curcumin-loaded CD-graphene oxide core (Cur@CD-GO) and gallic acid-loaded chitosan shell nanofibers (Cur-Ga NF) using electrospinning [123]. The release characteristics of Cur and Ga from the Cur@CD-GO and Cur-Ga NF were evaluated at pH 5.4. The total amount of the curcumin released from Cur@CD-GO was 91% at 24 h, which reached 95% after 72 h, whereas in Cur-Ga NF these figures were 60% to 78%, at the same times. Notably, in the Cur@CD-GO system, almost 70% of curcumin was released within 12 h. Therefore, the results showed that curcumin releases from the Cur-Ga NF were slower than those of the Cur@CD-GO. Additionally, this nanosystem showed high potency against human alveolar epithelial cancer cells compared with single drug nanofibers, and a higher antibacterial activity evaluated in the Gram-positive bacteria Bacillus cereus and the Gram-negative bacteria *Escherichia coli*. Interestingly, the loaded nanofibers (Cur-Ga NF) showed high cell viability at low concentrations in the human epidermoid mouth carcinoma cell line KB (healthy cell). Although the cytotoxicity observed in Cur@CD-GO was associated with the presence of GO, it was deduced that the coating carried out increased the cell viability of this material.

In general, the synergistic binding between CD and GO increases the charging capacity, mainly due to weak interactions such as π–π, electrostatic, and supramolecular (host–host). The presence of a polymeric material also contributes to weak interactions and grants new functions; however, the amount of loaded drug does not always increase. Remarkably, these systems respond to multiple stimuli for drug release, with pH being the most frequent method, whereas GO-grafted CD improves dispersion in water and increases the stability and biocompatibility of GO in physiological media. There are no in-depth studies on the bioavailability of these new nanosystems.

On the other hand, recent studies on carbon-derived nanomaterials have accentuated carbon dots and graphene dots as new nanostructures for the diagnosis and treatment of diseases, since they have fluorescence properties and have shown lower toxicity, greater biocompatibility, chemical inertness, and higher aqueous solubility than semiconductor quantum dots. Typically, graphene dots have layered structures and a lateral size of up to 100 nm; that is to say, they are small graphene sheets. In contrast, carbon dots have a spherical shape with a diameter of up to 10 nm, which, together with their edge properties and biocompatibility, gives them an advantage in studies on drug transport and release [124].

Yang and col. fabricated a nanocarrier for DOX functionalizing carbon dots with CDs, for anticancer effect [125]. The loading capacity for DOX was 27% (at pH 7.4) and was mediated by interactions with the supramolecule. The ability of the fluorescent system to target folate-receptor-positive cells was confirmed, together with efficient intracellular uptake. The drug release of 82% was activated by a decrease in pH to values of 5.0. Remarkably, a fluorescence resonance energy transfer occurred between the carbon dots (donor) and DOX (acceptor), which could be useful in tracing the release process. These promising results demonstrate the advantages of the combined use of carbon quantum dots and CDs, which, besides transporting the drug, could monitor the pH-activated release event. Table 2 summarizes the important data on the aforementioned graphene derivative-based nanosystems.

### 2.5. Inorganic Nanoparticles

The versatility of native and modified CDs for drug delivery applications is also present in organic–inorganic hybrid systems. Specifically, CDs can be used to synthesize, stabilize, or modify inorganic nanomaterials. Next, the uses and new functions provided by incorporating polymers and CDs in the most relevant inorganic nanoparticles are exposed. A complete summary of the most important data can be seen in Table 2.

#### 2.5.1. Mesoporous Silica Nanoparticles

Silica-based nanoparticles in different forms such as solid, hollow, or mesoporous are considered non-toxic and biocompatible, and present relevant results in the area of drug delivery. Mesoporous silica nanoparticles are pioneers in this area, since they possess an adjustable size and highly ordered internal mesopores in a large volume. Its large surface area can be functionalized to design multi-functional systems with high loading capacity and controlled release [126]. Mesoporous silica nanoparticles do not have an intrinsic capacity to respond to stimuli; therefore, controlled drug release is achieved through chemical modification using polymers or supramolecular structures, among others. In this sense, the CDs derivatives are used as gatekeepers to prevent early release, or control the release, of loaded drugs in the mesoporous matrix, since host–guest interactions can respond to external stimulus signals, such as magnetic field, light, electricity, pH, redox, and temperature [127]. For their part, polymers have a versatile application, as they are used during synthesis to improve dispersion or modify the size of pores. In addition, they can be used as gatekeepers of the pores, controlling the release of drugs through stimuli and increasing the stability of the nanosystem.

For example, Bian and col. constructed a visible-light triggered drug binding and releasing system between tetra-ortho-methoxy-substituted azobenzene and βCD modified mesoporous silica nanoparticles [128]. The nanoparticles functionalized with amino-βCD were covered with the visible-light responsive copolymer Azo-PDMAEMA. Green light (520 nm) triggered the opening of azobenzene/βCD valves and the release of the drug p-Coumalic acid by 56%, reaching equilibrium after 30 min. A control experiment without irradiation showed that 7% of the drug was released even after 50 h, which may afford great potential for cancer therapy.

#### 2.5.2. Plasmonic Nanoparticles

Plasmonic nanoparticles have emerged as an excellent alternative to design new smart drug carriers, mainly due to their tunable optoelectronic properties, such as localized surface plasmon resonance. In addition, due to their high chemical reactivity, they can be functionalized with biological materials or chemical species that add new advantages not only in drug delivery, but also in the diagnosis and treatment of diseases, photothermal therapy, bio-imaging, and stimuli-responsive drug release, for example [129,130,131,132,133,134]. Plasmon nanoparticles have a wide range of shapes such as spheres, cubes, stars, prisms, rods, core-shell and hollows, among many others [135,136,137,138,139,140,141]. These nanosystems do not stand out for a high drug-loading capacity, and thus for this reason polymers, polymers modified with CDs, and polymers of CD are used. Additionally, these materials allow an improvement in biocompatibility, bioavailability, and colloidal stability [142].

Donoso and col. formed the first nanosystem based on gold nanostars and βCD cationic polymers [143]. The star shape of these anisotropic nanoparticles is highly reactive; therefore, the polymer increases colloidal stability due to steric effects and electrostatic interactions. In addition, it allows the simultaneous loading of two drugs, phenylethylamine, and piperine, for its potential application in theranosis for mental disorders. Loading efficiency varied between 91% and 76% when the concentration of both drugs increased from 1.25 to 5.0 mM, whereas loading capacity was 95% at the maximum concentration of the drugs added to the nanosystem. Celebioglu and col. used CDs as a component for the manufacture of nanofibers and as a reducing agent, at the same time, for the formation of silver nanoparticles, creating electrospun CDs/silver nanoparticles and nanofibers [144]. The antibacterial activity of these nanofibers was studied against Escherichia coli and Staphylococcus aureus during a 24 h treatment. Therefore, plasmonic nanoparticles are versatile materials for drug transport and as therapeutic agents.

CD derivatives can contribute to the synthesis of these nanomaterials as reducing and stabilizing agents. Notably, the controlled release of drugs can be achieved by applying an external stimulus, such as a laser, on the plasmonic nanoparticles, an aspect that is not achieved by using only CDs [2].

#### 2.5.3. Magnetic Nanoparticles

There is great diversity among magnetic nanoparticles, which can be made up of one or more metals with a magnetic nature; their forms can be solid, hollow, core-shell, or coated with a material such as silicon dioxide, mesoporous silica, natural or synthetic polymers, noble metals such as gold or silver, or other stabilizing molecules. Due to their magnetic properties, these nanoparticles are used in numerous bio-applications as magnetic contrast agents in magnetic resonance imaging, in hyperthermia treatments—where the nanoparticles are selectively heated by applying a high-frequency magnetic field—and as magnetic vectors that a magnetic field gradient can direct towards a specific site of action [145].

Commonly, magnetic nanoparticles are coated with polymers to increase their stability, give new functions in drug delivery, and decrease cytotoxicity, which is inherent to its composition. For example, Mrówczyński and col. used polydopamine to coat the DOX-loaded mono-6-thio-βCD functionalized magnetic nanoparticles [41]. The cellular internalization of the nanosystem was evaluated in liver cancer; an in vitro study showed that the polydopamine layer was able to absorb near-infrared light (808 nm) with high performance, generating a combined chemotherapeutic and photothermal treatment. With the incorporation of CD, the encapsulation efficiency of the nanosystem was 90%. The maximum release of DOX was reached close to 50 h, and was 9% and 10% at pH values 5.5 and 4.5 (pH of cancer cells), respectively. This shows that this nanosystem offers a slow and sustained release profile. As a control, the release of the drug was evaluated at pH 7.4, being 2% at 48 h.

Ramasamy and col. used a biocompatible CD–dextran polymer to coat nickel ferrite nanoparticles, and load the drug camptothecin to improve cancer treatment [146]. The loading capacity of the new nanosystem was 88%; in addition, a sustained release of the drug was demonstrated in an in vitro study for 500 min. The release rate increased with a change in pH from 7.4 to 6.0. The studies in cell lines showed the cytotoxic effect of the nanosystem, which was higher compared with cisplatin and fluorouracil antineoplastic.

#### 2.5.4. Quantum Dots

Quantum dots are ultra-small semiconductor nanoparticles with excellent optoelectronic properties, that are successfully applied in the biomedical area. They are synthesized from elements of group II and VI, III and V, or IV and VI, and obtained with a hydrophobic coating that must be modified to improve its aqueous dispersion. In such cases, the use of polymers is recurrent, reducing, in turn, the cytotoxicity of the nanosystem, especially if they are composed of heavy metals.

Quantum dots can fulfill multiple tasks for the treatment of diseases; for example, they can be loaded on the surface with drugs and directed to the site of action, while fluorescence allows local images to be obtained. Due to their size, it is common for quantum dots to be incorporated into other nanomaterials for drug delivery, such as hydrogels, mesoporous silica, polymeric nanoparticles, or liposomes. Additionally, they are sensitive to stimuli, since fluorescence can be altered by a change in pH or by changes in the chemical environment, which provides additional information on drug release [147]. Precisely, Shu and col. designed a new nanosystem, incorporating ZnSe/ZnS quantum dots on βCD and chitosan polymer loaded with suberoylanilide hydroxamic acid [148], where the drug encapsulation efficiency was 22%. The drug release efficiency in the tumor microenvironment (pH 5.3) was higher than that in the physiological pH 7.4. Additionally, in vitro cytotoxicity studies showed that the blank nanoparticles had no cytotoxicity, whereas the drug encapsulated in the nanoparticles expressed an anticancer effect.

In the field of metal nanoparticles (composed of gold, silver, iron, or others), the functionalization with CD derivatives is helpful to increase the loading capacity of drugs, however, they do not outperform other nanomaterials such as graphene or nanocarriers based on lipids or polymers. In any case, this functionalization improves colloidal stability and reduces their inherent cytotoxicity, giving them greater attributes for studies on bioavailability.

### 2.6. Other Nanosystems

As discussed, non-covalent and reversible interactions using CD derivatives have reached a broad spectrum of applications in drug delivery. Briefly, other studies are presented where CD derivatives play a relevant role in improving the physicochemical properties of drugs or nanomaterials, and are crucial to achieving controlled release and improving cell viability or bioavailability, of the different nanosystems presented. Table 2 summarizes data from recent articles, including other nanosystems.

#### 2.6.1. As Nanovalves

An alternative and interesting method of applying CD to different nanomaterials are through nanovalves. Nanovalves are CD-modified or grafted onto other molecules, and assembled to organic or inorganic nanostructures to block or unblock drug release. This action is triggered by applying a stimuli such as light irradiation, pH, thermal, redox, competitive binding, magnetic field, chemical signals, and changes in the biological environment [149]. For example, in a study carried out in 2018, the multiple cavities of mesoporous silica nanoparticles were covered with CDs and 2-diazo-1,2-naphthoquinone molecules, forming a gatekeeper or nanovalve [150]. Based on the hydrophobic to hydrophilic transformation of 2-diazo-1,2-naphthoquinone induced by NIR light, a hydrophilic product was generated. which allowed dissociation of the supramolecular assembly and subsequent release of DOX on demand for anticancer effect.

#### 2.6.2. Metal–Organic Frameworks

Metal–organic frameworks (MOF) are among the most researched materials in contemporary chemistry. They are composed of organic linkers and metals of the transition and lanthanides groups, obtaining a crystalline and porous material through coordinated bonds [151]. The applications are diverse, such as the synthesis of metal nanoparticles, catalysis, and drug delivery. CDs have adhered to these systems to form new biocompatible materials. As additional results, they increase drug stability and aqueous solubility, and improve loading capacity, since CD molecules do not compete to enter their porous structure. Consequently, in vivo studies have been performed to improve the bioavailability and absorption rate of active ingredients [152].

Following the same line, MOF nanoparticles have emerged as new systems; however, they require much exploration due to their high chemical reactivity, and new properties intrinsic to their nanometric dimensions [153]. Agostoni and col. functionalized MOF nanoparticles with phosphated CDs using iron (III) polycarboxylates to form a biodegradable material [154]. Uncoated MOF nanoparticles are usually unstable in aqueous solutions; however, interactions with modified CDs improve their stability, even in microenvironments containing competing phosphates. This was determined in cell culture medium and phosphate buffer solution at pH 7.4, for times greater than 24 h. Added to this, it was shown that they are not toxic to the conditions studied. The phosphate forms of gemcitabine and azidothymidine triphosphate were used as model drugs (for potential therapy of cancer and immunodeficiency syndrome, respectively), and it was shown that the presence of phosphate CDs did not interfere with entrapment and release. Finally, the intracellular uptake of the nanosystem was evaluated. For this, the MOF nanoparticles were modified with a CD-phosphate derivative containing mannose. The amount of quantified iron in the human retinoblastoma cell line Y79 (which overexpressed the mannose receptor), was more than double, compared with the system without modified CD.

Remarkably, CD derivatives open a new path of applications for MOF nanoparticles in drug delivery, since they can form multifunctional systems, increasing their biocompatibility without affecting this nanomaterial’s high intrinsic-loading capacity.

#### 2.6.3. Janus Nanoparticles

Janus nanoparticles are smart materials composed of two or more different structures of different natures. Due to their anisotropy, they present unique properties, and if properly designed they can be used as biocompatible nanocarriers for drugs [155]. Janus nanoparticles based on mesoporous silica and gold nanostars were prepared, which were capable of releasing loaded molecules after irradiation with NIR [156]. The surface of the gold nanostars functioned as a photochemical transducer and was functionalized with a thiolated photolabile molecule, whereas the mesoporous silica nanostructure was loaded with DOX and blocked with proton-sensitive benzimidazole-βCD supramolecular gatekeepers. The designed Janus nanoparticles were not cytotoxic to HeLa cells until they were irradiated with an NIR laser, leading to the release and intracellular hyperthermia of DOX, for potential anticancer effect. This induced a marked reduction in the viability of HeLa cells.

#### 2.6.4. Nanofibers

Nanofibers have been used to improve the administration of poorly soluble drugs, mainly orally. These have a large specific surface area and are functionalizable to increase loading and personalized and controlled drug delivery, among other advantages. In this sense, CDs are a functional material that have been incorporated in multiple works, increasing stability and bioavailability without interfering with fiber degradation [6].

Polyurethane nanofibers, with and without βCD, were formed to increase the antibacterial activity of gentamicin, using a modified electrospinning method. The functionalization with CDs improved the physicochemical properties of the nanofibers, such as tensile strength and thermal stability, in addition to exhibiting blood biocompatibility, cell viability, and a greater release of the drug concerning non-functionalized nanofibers. Furthermore, this polyurethane–CD system at optimal conditions (15 wt% CD) was loaded with gentamicin, and exhibited the best antibacterial properties against Gram-positive and Gram-negative bacteria [157].

**Table 2:** Summary of the most important data based on the qualification according to the type of nanosystem, type of CD used, types of polymers, and results regarding drug loading, release mechanism, and in vivo studies (if available) from Section 2.3, Section 2.4, Section 2.5 and Section 2.6.

	System	CD	API	Loading Capacity	Loading Efficiency	Drug Release Mechanism	In Vivo Studies	Ref.
Polymeric nanosystems based on CDs	βCD polymer-based nanosponge	βCD	Temoporfin	-	-	Diffusive release under tumor spheroid conditions	-	[97]
	βCD polymer-based nanocarrier	βCD	Sorafenib	~5.7 and ~9.9 mg/g	-	Diffusive release under cell and mice pshysiological conditions	Toxicity and accumulation	[98]
	βCD-based polymer with RGD peptides	βCD	DOX	-	-	Diffusive release under carcinogenic cellular conditions	-	[99]
	Cationic βCD polymer with fluorescent probe nanocarrier	βCD	Diclofenac	16%	100%	Diffusive release under physiological conditions	-	[100]
	βCD polymer-based nanoparticle	βCD	Ethionamide and BDM-smart420-booster	~21 and ~6 mg/g	-	-	-	[102]
				25 mg/g	-	Diffusive release in vivo	Efficacy	[101]
	Pseudopolyrotaxane-βCD-based polymer	βCD	Novobiocin	23%	-	Diffusive release under physiological conditions	-	[103]
			Vancomycin	6%	-			
			Novobiocin	18%	-			
			Vancomycin	6%	-			
	βCD-based nanosponge tablet	βCD	Paracetamol, aceclofenac and caffeine	-	81–89%	Diffusive release from the tablet	-	[104]
	βCD-based nanosponge suspension	βCD	Nifedipine	-	78%	Diffusive release under simulated gastric fluid	Oral bioavailability	[105]
	βCD polymer-based nanosponge	βCD	Atorvastatin	-	34%	Diffusive release in dialysis sac methods	Oral bioavailability, pharmacodynamics, and efficacy	[106]
	βCD-based nanosponge tablet	βCD	Febuxostat	-	88–100%	Diffusive release using dissolution apparatus	Oral bioavailability	[107]
	βCD polymer-based nanosponge functionalized with gold nanoparticles	βCD	Phenylethylamine	90%	-	-	-	[110]
			2-amino-4-(4-chlorophenyl)-thiazole	150%	-			
Graphene derivatives	Multi-walled carbon nanotubes CD-Maleic Anhydride-N-Isopropylacrylamide-Fluorescein-folic acid	βCD	Curcumin + DOX	29 wt% (curcumin) and 19 wt% (DOX)	92%	Temperature change, pH change and laser irradiation at 808 nm	Progression and regression of tumor in BALB/c mice model	[116]
	Graphene oxide-L-phenylalanine-βCD	βCD	DOX	85%,	79%	pH change	-	[118]
	Graphene oxide-Fe_3_O_4_-βCD	Mono-6-deoxy-6-ethylenediamino-βCD	DOX	4%	37%	pH change	-	[119]
			Methotrexate	2%	23%			
	Graphene oxide-βCD-poly(amido amine) dendrimer	aminated-βCD	DOX	0.4 mg/mg	-	pH change	-	[120]
			Camptothecin	4.0 mg/mg				
			Protoporphyrin IX	0.8 mg/mg				
	Graphene oxide + mPEG−QPDMAEMA/α-CD supramolecular hydrogel	αCD	5-fluorouracil	4.6 mg/g	-	Temperature change, pH change and UV irradiation responsive release	-	[121]
	βCD/Ni nanoparticle-modified GO and mitochondrial ion-targeting peptide-grafted hyaluronic acid	Mono-6-deoxy-6-ethylenediamino-βCD	DOX	>36%	-	AMF responsive release	-	[122]
	Core: Curcumin@CD-oxide graphene, Shell: Gallic acid@Chitosan	6-O-monotosyl-βCD	Curcumin			pH change	-	[123]
			Gallic acid					
	βCD/Carbon dots	βCD	DOX	27%	-	pH change	-	[125]
Associated to inorganic nanoparticles	Tetra-ortho-methoxy-substituted azobenzene/βCD-modified mesoporous silica nanoparticles	aminated-βCD	p-Coumalic acid	-	-	Green light (520 nm)	-	[128]
	Gold nanostar modified with cationic βCD-based polymer	βCD	Phenylethylamine and piperine	95%	91–76%	-	-	[143]
	Electrospun CD/Ag nanoparticles nanofibers	HPβCD	Ag nanoparticles	-	-	ions on agar plates	-	[144]
	Fe_3_O_4_ magnetic nanoparticles functionalized with loaded mono-6-thio-βCD	6-thio-βCD	DOX	90%	-	pH change	-	[41]
	Nickel ferrite nanoparticles covered with CD-dextran polymers	Mono-6-deoxy-6-aminoethylamino-βCD	camptothecin	-	-	pH change	-	[146]
	ZnSe/ZnS quantum dots on βCD/chitosan polymer	βCD	Suberoylanilide hydroxamic acid	22%	-	pH change	Biodistribution in a melanoma animal model injected subcutaneously	[148]
Other nanosystems	Mesoporous silica nanoparticles modified with CDs/2-diazo-1,2-naphthoquinone nanovalves	βCD	DOX	5%	69%	NIR light irradiation	Intratumoral injection in tumor-bearing mice	[150]
	MOF nanoparticles functionalized with iron (III) polycarboxylates/CDs	Phosphated CD	Azidothymidine-triphosphate	8%	-			[154]
	Janus gold nanostar–mesoporous silica nanoparticle modified with a thiolated photolabile molecule and proton-responsive benzimidazole-βCD	β-CD	DOX	-	-	NIR light irradiation	-	[156]
	βCD functionalized polyurethane fibrous membranes	βCD	Gentamicin sulphate	68%	-	Diffusive release in PBS at pH 7.4	Antibacterial activity against Gram positive Staphylococcus aureus and Gram negative Escherichia Coli	[157]

## 3. Cyclodextrin Derivates for Stimuli-Controlled Drug Release

A formulation based solely on CD derivatives can enhance the pharmacological and physicochemical properties of the drugs used for biomedical applications [158,159], simultaneously enhancing loading capacity, aqueous solubility, bioavailability, and delivery to the target site. Nevertheless, these systems lack an intrinsic mechanism that controls the drug release; therefore, efficient administration to a specific site depends on the host–guest interactions and the biological microenvironment of the pathways. In this sense, the combined use of CD derivatives with polymers and nanomaterials can avoid nonspecific release, and control the administration of drugs by application of internal (pH, reductant environments, enzymes) and external (light, magnetic fields, ultrasound) stimuli [33]. 

Therefore, the drug delivery mechanisms of CD-modified nanomaterials depends on the location of the inclusion complexes in the nanosystem and the characteristics of the components of the nanomaterial. Typically, the drug release from inclusion complexes can occur from the dilution of the complex, competitive displacement of the drug against molecules with a higher affinity for the CD cavity, drug binding to plasma and tissue components, uptake of the drug by tissues not available to the complex or CD, CD elimination, and possibly pH and temperature adjustments [160]. These processes dominate when the complexes are bound covalently, or by weak interactions with the surface of the nanomaterial, as is the case with nanosponges or some solid core nanosystems, such as metal nanoparticles.

On the other hand, there are nanosystems where the complexes are included within the nucleus or matrix of the nanomaterial, as in lipid or polymeric nanoparticles. In these cases, the main drug release mechanisms are diffusion, erosion, and swelling [160], depending on the composition of the nanomaterial; for example, those systems made up of pH-sensitive polymers release the complex or drug in specific ranges or values of acidity or basicity.

In this regard, the use of thermosensitive materials for drug release, which change their physical properties at a physiological temperature, have been explored. For example, Okubo et al., 2020, synthesized a thermo-responsive injectable hydrogel for diabetes treatment, based on hydrophobically modified hydroxypropylmethylcellulose/βCD [161]. This hydrogel was able to change its viscosity in response to the body temperature, due to the host–guest interaction between the stearyl moieties of cellulose derivate and the βCD cavity, for the sustained release of the drug. Interestingly, the proposed hydrogel became a gel at body temperature (37 °C), and increased the circulation time of indocyanine green (ICG, drug model) and insulin when subcutaneously administered to a mouse model. Insulin administered in the hydrogel formulation showed a prolonged hypoglycemic effect in response to the insulin, slowly released from the hydrogel. Nevertheless, more specific drug release could be achieved when the formulation has a thermosensitive behavior at a higher temperature, for example, in tumor or inflammation sites in cancer, where the local temperature reaches 42 °C [162]. In this regard, responsive nanoparticles fabricated from mesoporous silica and βCD-poly(*N*-isopropylacrylamide) were proposed by Guo et al. for the release of DOX [163]. Interestingly, these nanoparticles presented a 93% drug release at 42 °C, and showed a release gathered by H_2_O_2_, which is used as a cell stress biomarker.

The thermosensitive drug release strategy can be combined with another stimulus, such as pH at physiological or pathological conditions. For example, Lou et al. developed dialdehyde-βCD cross-linked carboxymethyl chitosan hydrogels for phenolphthalein release as a model drug for cancer drug delivery [164]. The hydrogels presented good release properties, 50% release at 2 h and 90% within 12 h, compared with only glyoxal/chitosan loaded, which released only 19% of the drug after 24 h, at physiological conditions (pH = 7.4, T = 37 °C). Additionally, Mamidi et al. proposed a hybrid supramolecular hydrogel based on poly (*N*-(4-aminophenyl) methacrylamide)) carbon nano-onions (PAPMA-CNOs = f-CNOs) and γ-CD/DOX-complex reinforced gelatin methacryloyl/f-CNOs/CD [159]. Such composites demonstrated improved thermal, wettability, swelling, and mechanical properties, and enhanced DOX drug release under acidic conditions (pH 4.5 = 99%, and pH 6.0 = 82%) compared with physiological pH (pH = 7.4), over 18 days. Furthermore, these nanocomposites showed to be biocompatible in human fibroblast cell lines. It is important to remark that this range of pH offers future applications in cancer drug delivery, since the acidic pH of the microenvironment of cancer cells (pH 4.5−6.5) differs from the pH of normal cells (pH 7.4).

In another study, Maqbool et al. prepared a celecoxib-βCD inclusion complex loaded Eudragit^®^ S100 (a combination of methacrylic acid and methyl methacrylate) microparticles for chronotherapy of rheumatoid arthritis [165]. In this work, colonic drug administration was approached due to their enhanced retention time, compared with the stomach and small intestine drug absorption. The nanoparticles had a drug entrapment efficiency of 68.47% to 91.65%. In vitro release pattern of the drug was assessed in simulated gastric and intestinal fluids. Drug release was initially delayed at the gastric and intestinal pH (pH = 1.2 and pH = 6.8, respectively), followed by a fast release at colonic pH (pH = 7.4) with first order release kinetics. This novel study provides the opportunity for oral administration of drugs, with colonic absorption after 5 h of administration.

On the other hand, a smart stimulus, such as specific biomolecules overexpression, is also used to trigger drug release. For example, this can be seen in studies such as those reported by Wang et al. [8] and Xu et al., in which modified βCD-loading insulin triggered release in the presence of high glucose concentrations [166,167]. In both studies, the formulations were demonstrated to be safe and specific glucose-responsive drug carriers with potential use for the therapy of diabetes.

In addition to internal stimuli-responsive strategies, external-stimuli strategies can boost the therapeutic properties of the nanocarriers. In this regard, Wu et al. reported a novel light/redox-sensitive material for cancer therapy, based on mesoporous silica nanoparticles functionalized with an azobenzene/galactose-grafted polymer and βCD for DOX release [168]. In this work, DOX was released due to the dissociation of azobenzene moieties from the βCD cage after UV-irradiation, followed by the reductant effect glutathione, as an endogenous metabolite. The redox and UV-sensitive DOX release was confirmed in vitro. Interestingly, the DOX@MSN-ss-CD/GAP demonstrated enhanced cytotoxicity into HepG2 cells, compared with HeLa and COS7 cells. In other studies, electromagnetic fields as external stimuli, and pH as an internal promoter, have been used to control triggered drug release. For example, Enoch et al. synthesized a novel pH-sensitive magnetic hydrogel from carboxymethyl cellulose, βCD, and chitosan, combined with Magnetic Fe_3_O_4_ nanoparticles, for the controlled release of MTX with a release of 93% at pH 7.4 in the presence of an AMF potentially useful for cancer therapy [169].

**Figure 3:**
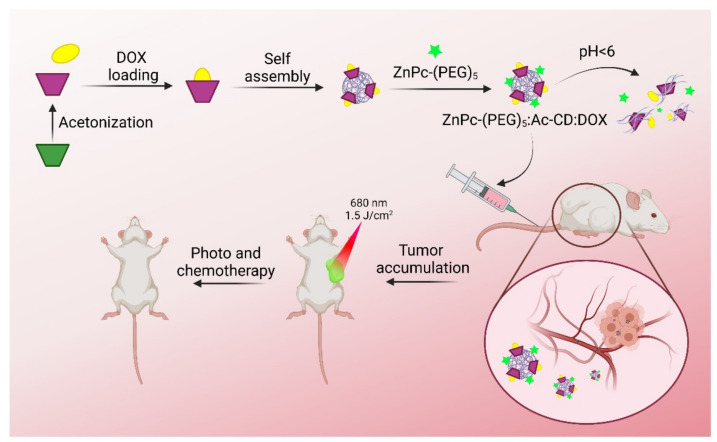
Construction of the nanosystem ZnPc-(PEG)_5_:Ac-CD:DOX with pH and light sensitivity for in vivo synergistic chemo- and photo-therapy of tumors in an H22 tumor-bearing mice model [170]. Created with BioRender.com.

In a recent study published by Zheng et al., the suitability of materials combined with CDs for externally and internally triggered drug release for in vitro and in vivo photothermal therapy was established [170]. As summarized in Figure 3, βCDs were modified by acetonization (Ac-CD), loaded with DOX, and self-assembled to nanoparticles by emulsion–solvent evaporation methods. These nanoparticles were coated with a zinc phthalocyanine photosensitizer ZnPc-(PEG)_5_ for cancer therapy under laser irradiation with a 680 nm light source at 1.5 J/cm^2^. Additionally, the final product was demonstrated to have pH-sensitive drug release (pH = 6) and a synergistic cytotoxic effect of chemotherapy (by DOX) and phototherapy (by laser irradiation) in HepG2 cells. Interestingly, the nanoparticles had an effective tumor targeting, prominent antitumor activity, and fewer side effects in vivo in an H22 tumor-bearing mice model, due to 3 min irradiation in the tumor area (680 nm, 1.5 J/cm^2^) every 24 h for 7 days.

Stimulus-controlled drug release using nanomaterials in an outstanding method in biomedical research. The strategy of using smart nanosystems that are activated by both internal and external stimuli has been increasing, since it allows for personalized therapy and focuses on targeting the malignant tissues, instead of healthy ones. The combination of CD-based nanoformulations and stimuli-responsive nanomaterials has been explored for in vitro and in vivo approaches; however, it has not yet been explored in the clinical area.

**Figure 4:**
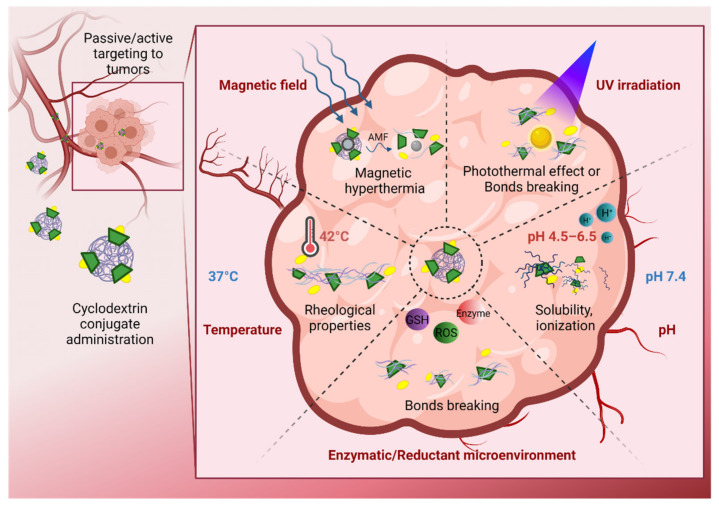
Stimuli-controlled drug release from CD-modified nanomaterials in tumor sites. The conjugates reach the tumor sites by passive/active targeting, then an external stimulus (alternating magnetic field, AMF, or UV irradiation) and/or an internal stimulus (temperature, intracellular metabolites, or acidic pH) triggers the drug release from the conjugates. Created with BioRender.com.

Most of the stimuli-triggered drug release studies focus on cancer treatment, due to their characteristic hallmarks compared with healthy tissue, such as an increased temperature, acidic pH, reductant environments, and the presence of specific enzymes/metabolites [171,172]. In Figure 4, the stimuli driven drug release for cancer treatment using polymer-CD conjugates is depicted. Increased temperature in tumor sites could be exploited to modify the rheological properties of the polymers and release drugs [161]. On the other hand, the acidic pH of the cells promotes changes in the solubility of the polymer-CD conjugates, and triggers the drug release, or the presence of reductant agents, such as glutathione (found in increased concentrations in tumor sites), could promote bond-breaking (mainly S-S bonds) and drug release in the target site [159]. In addition, externally controlled laser irradiation and ultrasound waves improve drug release strategies, mainly by combining with a photosensitizer or magnetic agents, increasing the local temperature and promoting bond-breaking and cell death by hyperthermia [168,169].

The design of stimuli-driven drug release materials is a challenge in itself. Materials should adhere to biosafety and be sensitive enough to respond to slight temperature, pH, or metabolite concentrations changes around the physiological conditions [33]. The main limitations for bringing these therapies into clinical models are related to the individual biochemical differences in the patients (blood composition, tumor heterogeneity, heterogeneity of the population), limiting a system from having the same controlled release effectivity in all patients [39,173]. Therefore, the more promissory strategies are those related to external stimuli-controlled delivery, combined with physiologically triggered ones. However, those external stimuli sources, such as laser irradiation or magnetic fields, are limited due to their low penetration in tissue (~10 mm) and their scattering on soft tissues. In addition, the possible side effects in the long term on the healthy tissues must be evaluated [174].

## 4. Bioavailability of Cyclodextrin-Modified Nanomaterials

**Figure 5:**
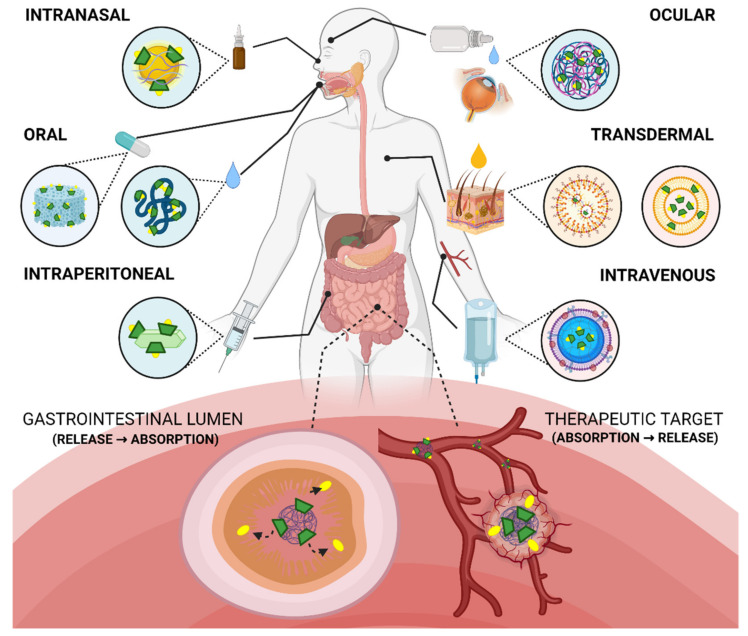
Different routes of administration where CD-modified nanomaterials have been shown to increase bioavailability. Created with BioRender.com.

Nowadays, approximately 40% of marketed APIs and 70–90% of new therapeutic compounds are very poorly soluble, and have permeability problems, with consequent absorption and bioavailability issues [175,176]. As a result, nanoparticles, CDs, and polymers are being widely studied to solve these problems and, as observed in Table 1 and Table 2, 32% of revised publications in this article include bioavailability studies by different administration routes (Figure 5).

Several factors can strongly impact drug bioavailability depending on the route of administration, such as the presence of biological barriers or physiological clearance processes. The combination nanoparticles and CDs can overcome these challenges facilitating the drug loading into the system, increasing absorption or passage through bio-barriers, and transporting the API to its therapeutic target. For example, transdermal administration implies crossing the epidermal/dermal drug barrier: the stratum corneum, the top layer of the skin, provides the main barrier to skin absorption. As discussed above, Mura et al. developed deformable liposomes to improve the anesthetic efficacy of transdermal Butamben, bearing the drug as a CD complex [58]. In vivo experiments on rabbits proved that liposomal formulations significantly increased the intensity and duration of drug anesthetic action, compared with its hydroalcoholic solution. Additionally, Pires et al. demonstrated follicular accumulation and the controlled delivery of *Lippia origanoides* essential oil, encapsulated in HPβCD inclusion complexes and included in NLC [74]. The results suggested that the developed system of follicular accumulation formed a depot for controlled delivery with a shelf life of 6 days.

On the other hand, the drug bioavailability after ocular administration is affected by the rapid washout of the drugs, due to nasolacrimal drainage or ophthalmic barriers. These drawbacks can be avoided by prolonging retention time in the cul-de-sac, or by increasing ocular permeability [177]. Maged et al. developed an econazole formulation based on the combination of HPβCD with Tween 80. The combination of HPβCD with Tween 80 prevented the econazole nanosuspension aggregation during storage and enhanced drug release from the nanosuspension. This antifungal nanoformulation was supplemented with chitosan-HCl to increase drug release and ocular bioavailability, demonstrating that the incorporation of polymers in this type of nanosystems is a substantial contribution [89]. Due to its polymeric nature and positive charge, chitosan increases viscosity and mucoadhesion, respectively, providing a longer contact time between the formulation and the eye. Nanosuspensions with chitosan have shown a higher concentration of econazole in tears over 7 h (122.5 μg/mL), compared with that for nanosuspension without chitosan (67 μg/mL) and drug suspension (52.8 μg/mL), which remained detectable for only 4 h after ocular administration.

Sukumar et al. explored the potential of the nose-to-brain direct transport pathway to bypass the blood–brain barrier, and to enable targeted delivery of theranostic polyfunctional gold–iron oxide nanoparticles (polyGIONs) in glioblastoma patients, through the intranasal route to sensitize glioblastoma cells to temozolomide, a chemotherapy drug systemically administered. The authors synthesized GIONs coated with a hybrid polymer (βCD-chitosan), co-loaded with miRNAs (miR-100 and antimiR-21), and covered their surface with adamantane-PEG-T7 peptide [178]. The resulting polyGIONs showed efficient miRNA loading, efficient accumulation of Cy5-miRNAs in mice, and a significant increase in the survival of mice co-treated with T7-polyGIONs loaded with miRNAs and temozolomide, compared with the other groups (untreated, non-targeted polyGIONs-miR-100/antimiR-21-treated, or temozolomide-treated mice). This novel theranostic intranasal nanoformulation demonstrates a strong potential to improve the effects of temozolomide treatment in glioblastoma patients.

An oral administration drug requires passage through the gastrointestinal tract. Undoubtedly, this route is one of the best options because it is non-invasive, making it worth exploration. Nanosystems designed for the oral route can be divided into two categories: (1) nanoparticle systems intended to only increase the dissolution rate of an API (increase absorption and bioavailability); (2) nanoparticle systems intended to be absorbed as designed and for the drug to be delivered once the system has crossed the intestinal barrier. The former must have a significant advantage compared with simpler pharmaceutical systems, composed of well-known and approved excipients, to be considered in further studies. The second needs to prove that it has not degraded before reaching its target. In this sense, a very interesting study including the analysis of size, surface charge, and hydrophobicity/hydrophilicity of nanoparticles for oral administration was recently published [179]. Authors have found that smaller size (in a certain range), low-magnitude negative charge, and moderate hydrophilicity can help complete the process of transintestinal epithelial cell transport.

Thus, an oral drug with low bioavailability would require large doses to reach effective concentration after absorption [180]. As discussed, bioavailability studies have shown that nanosponges loaded with febuxostat [107], nifedipine [105], or atorvastatin [106], increase pharmacokinetic parameters two to four times, compared with references after oral administration. As expected, nanosponge’s cavities facilitate drug entrapment and slow release, leading to mean residence time, or a delayed Tmax as shown in Amin et al., 2020, and Zidan et al., 2018 [106,107]. On the contrary, a drug administered intravenously is assumed to be immediately delivered to the systemic circulation. For example, Song et al. evaluated the bioavailability of paclitaxel and IL-2-loaded nanogels formulated with HPβCD-acrylate and chitosan derivatives for combinatorial effects of chemotherapy and immunotherapy in cancer treatment. The plasma paclitaxel and IL-2 concentrations were measured after IV injection (4 mg/kg paclitaxel and 2.5 μg/kg IL-2). In comparison with free paclitaxel + IL-2, nanogels showed respective 2.2-fold paclitaxel and 2.6-fold IL-2 concentration at 0.5 h, and the circulation time of IL-2 was also longer. Non-coated nanogels demonstrated a 4.6-fold increase in the area under the curve (AUC0−t), compared with those of free paclitaxel + IL-2. Moreover, the red blood cell membrane-coated nanogels exhibited a higher half-life than those of non-protected nanogels, attributed to the biomimetic structure that makes them more biocompatible and stable [83].

Enhancing drug bioavailability would lead to a decrease in the required dose to reach the pharmacological concentrations, which ultimately translates to optimized treatments, minimized adverse events, and improved patient compliance. Caillaud et al. demonstrated that curcumin-CD/cellulose nanocrystals reached a higher average plasma concentration of curcumin at t = 1 h (26.3 versus 24.9 ng/mL, respectively) and t = 2 h (5.3 versus 4.3 ng/mL, respectively), even at a dose 250 times lower than that of free curcumin (0.2 mg/kg versus 50 mg/kg), demonstrating a strong enhancement in curcumin’s bioavailability [79]. Additionally, nanocrystals showed a prolonged release of curcumin in plasma for at least 12 h after intraperitoneal administration (detectable until 72 h post-injection), whereas free curcumin was not detectable after 8 h. This outstanding enhancement in bioavailability is reflected in the demonstrated superior efficacy through a large number of in vivo experiments on Charcot-Marie-Tooth-1A transgenic rodents [79].

For any administration route, vectorization of a nanosystem to target a specific site would ideally increase the drug concentration at that site of action, improving efficacy and minimizing adverse events. Some of the research articles described in this review have investigated the drug levels in different tissues to evaluate the targeting capability of the nanosystems [58,74,79,83,90,148,178]. Considering dose and administration route, computational methods could be able to predict the bioavailability of any kind of formulation including complex nanosystems. Although the current works focus on simple pharmaceutical systems, in silico predictions as well as high-throughput experiments show promise in the pharmaceutical field in reducing development timelines and costs [181]. Unfortunately, most in silico studies are focused on new compounds [182,183] rather than new excipients/carriers/pharmaceutical systems [184], losing the benefits of drug repositioning [185] or the potentiality of improving the bioavailability of marketed compounds towards the ideal formulation (i.e., all active molecules reach their target).

Ultimately, as a limitation of the research studies summarized in this review, it is important to mention that the majority of them show data from formulations with a single API as a drug model [175]. It would be interesting to study the versatility of the nanocarriers in enhancing the bioavailability of different APIs.

## 5. Final Remarks

The chemical modifications made to CDs have allowed constant innovation and the incorporation in all kinds of nanosystems for drug administration. Among these, polymerized, cross-linked, and/or nano-sized CDs have been discussed, which are attached or loaded to lipid-based nanocarriers, polymers, inter-unit polymerization forming a network, nanomaterials, or a more complex nanosystem. Through these processes, it is possible to obtain an advantage of a higher loading efficiency by the formation of new cavities, due to crosslinking, blocking the exit of the guest, or acting synergistically with the nanomaterial. In addition, the solubility and/or stability of the guest can be improved; at the same time, early release and toxicity in non-specific sites can be avoided. It is important to note that by modifying their composition, shape, and dimensions, the physicochemical characteristics of the different nanocarriers can be conveniently tuned for different biological scenarios or disease. In this regard, an important goal of these modifications is to achieve slower clearance times from the bloodstream and higher absorption, improving the bioavailability of API, which is essential for application during in vivo administration. Remarkably, the current research allows us to infer that CD-based nanomaterials will increasingly target the design of smart materials that enable several or even all of the aforementioned concepts.

Despite the advantages demonstrated by hybrid nanoparticle-CD systems, and the wide spectrum of diseases that can be treated, especially cancers, at the moment there are no products of this type on the market. Researchers explore new avenues that can potentially change paradigms or solve unmet needs. However, in most cases, novel systems do not seem to be worth carrying out in vivo and clinical studies. Certainly, clinical trials are mostly focused on new molecules, whereas those comparing excipients are quite low. It will take time to see nanocarrier excipients as the ones studied in this review in clinics. In this point, several aspects must be considered. On the one hand, many of the APIs studied applying this strategy have low solubility and/or low permeability, and therefore low bioavailability. This means that the recommended doses of these drugs are usually high. This generates a problem related to the fact that the formation of complexes with CDs usually uses, minimally, equimolar drug:CD ratios (1:1). The molar weight of the native CDs, which give rise to most of the derivatives studied, is 972 g/mol for αCD, 1135 g/mol for βCD, and 1297 g/mol for γCD. Additionally, the weight corresponding to the other components of the nanosystem has to be considered, so achieving the recommended doses of these drugs would imply the administration of a large amount of pharmaceutical formulation, hindering the patients’ compliance. Due to this fact, loading capacity becomes a key factor in the design of hybrid nanoparticle–CD systems, and only some nanomaterials would meet that requirement. In vivo effectiveness studies should demonstrate a decrease in the dose needed due to bioavailability improvement. On the other hand, it should be considered that the approval periods for this type of development are longer than those of the inclusion complexes that have already been approved as safe excipients for the pharmaceutical formulation. In addition, scaling processes for the formulation of nanosystems, from the laboratory to the industrial scale, reduce the application of these technologies, as they are more complex and expensive. Consequently, this new approach should be reserved for purposes that cannot be overcome with formulations manufactured by conventional methods.

Finally, it is worth mentioning that the pharmaceutical field has the role of creating new nanocarriers and pharmaceutical formulations, but it should not lose sight of the main goal of improving bioavailability and efficacy, while keeping side effects at a minimum. Building interdisciplinary teams constituted by researchers who think from different points of view and on different Innovative and Design projects stages would aid in the design of carriers with great potential translation into clinics.

## 6. Conclusions

The versatility of CDs to be modified has positioned them as one of the main supramolecular structures applied to drug delivery research; especially, when they are incorporated into different nanomaterials, whether these are classic materials such as lipid and metal nanoparticles, or recently discovered nanomaterials such as metal–organic frameworks nanoparticles and carbon dots. From a chemical point of view, CD derivatives stand out for improving the aqueous solubility and stability of drugs; however, combined with nanomaterials they can provide the ability to increase loading efficiency, improve colloidal stability and even obtain multifunctional nanosystems, among other advantages. Additionally, the controlled release of drugs triggered by a wide spectrum of stimuli (internal or external) is possible, bringing us closer to specific and personalized therapy. From a biological point of view, CD derivatives have been used to increase the bioavailability of drugs and nanosystems, decrease cytotoxicity and improve biosafety, as has been seen in different in vivo and in vitro studies. Undoubtedly, the versatility of modified CDs and their synergy with nanomaterials will allow them to continue expanding, not only in the field of supramolecular chemistry, but also in pharmaceutical sciences, and nanotechnology applied to biomedicine.

## Data Availability

Not applicable.
